# Modelling Hen Harrier Dynamics to Inform Human-Wildlife Conflict Resolution: A Spatially-Realistic, Individual-Based Approach

**DOI:** 10.1371/journal.pone.0112492

**Published:** 2014-11-18

**Authors:** Johannes P. M. Heinonen, Stephen C. F. Palmer, Steve M. Redpath, Justin M. J. Travis

**Affiliations:** Institute of Biological and Environmental Sciences, University of Aberdeen, Aberdeen, United Kingdom; University of California Irvine, United States of America

## Abstract

Individual-based models have gained popularity in ecology, and enable simultaneous incorporation of spatial explicitness and population dynamic processes to understand spatio-temporal patterns of populations. We introduce an individual-based model for understanding and predicting spatial hen harrier (*Circus cyaneus*) population dynamics in Great Britain. The model uses a landscape with habitat, prey and game management indices. The hen harrier population was initialised according to empirical census estimates for 1988/89 and simulated until 2030, and predictions for 1998, 2004 and 2010 were compared to empirical census estimates for respective years. The model produced a good qualitative match to overall trends between 1989 and 2010. Parameter explorations revealed relatively high elasticity in particular to demographic parameters such as juvenile male mortality. This highlights the need for robust parameter estimates from empirical research. There are clearly challenges for replication of real-world population trends, but this model provides a useful tool for increasing understanding of drivers of hen harrier dynamics and focusing research efforts in order to inform conflict management decisions.

## Introduction

The relationship between large predators and humans has always been a difficult one [1], [2]. Predators either threaten human life or compete with humans over shared resources and, as a consequence, a common response has been to try and remove them from ecosystems (e.g., [3], [4]). Such action often brings land managers and local communities into conflict with conservation practitioners who seek to protect these often rare and threatened species. Finding effective ways of dealing with such conflicts is increasingly recognised as an important challenge for conservation [5], [6].

One of the challenges to the development of effective management strategies for predators lies in understanding spatial variation and predicting what might happen under different scenarios [7]. In the absence of any human interference, we would expect the abundance of predators to vary between areas in relation to, among other factors, food abundance and habitat. An understanding of this variation is valuable in predicting where the impact of predators on prey of human interest is likely to be greatest and therefore where the often limited conservation resources should be focused.

There have recently been attempts to map risk and conflict. These have utilised advances in computing technology and tools such as geographical information systems to combine diverse spatial data as predictors of risk [8]. Explanatory power of predictors has been analysed using various statistical methods, including univariate and multivariate logistic regression [9]–[11] and machine-learning algorithms such as artificial neural networks [9]. In other cases, scientific findings and expert opinion have been relied on to identify important predictors and combined with analytical modelling for the extrapolation of factors across the landscape [8]. Examples include the development of spatial models to predict where predation of livestock by wolves *Canis lupus* (L.) is most likely to occur in Wisconsin and Minnesota, USA [11], [12] and maps of probabilities of human-black bear *Ursus americanus* (Pallas) interactions in Montana, USA [10]. Where data on predictors of spatial patterns in wildlife and human activity are available, these models can produce strategic management recommendations which can aid human-wildlife coexistence [9], [10]. However, it is important to consider the biology and movement behaviour of organisms being simulated when producing risk maps [11], which these types of models do not do.

Individual-based models (IBMs) have become popular in recent years in the fields of ecology and evolution (e.g., [13], [14]). Such models enable demographic processes to be represented based on the ecology and behaviour of the study species and to incorporate individual variability [15]–[17], thus allowing exploration of the effect of local mechanisms on population trends and spatial pattern formation and *vice versa* [12], [15]. In the conflict context, IBMs have been previously developed to investigate the effect of control practices on *Aedes aegypti* (L.) mosquito populations [18], voluntary mechanisms and farmers’ decision-making on land-use changes [19], [20] and the responses of animal populations to anthropogenic disturbance [21]. Hence IBMs have already become established in this field and there is potential for using them in understanding the spatio-temporal dynamics of conservation conflicts, potentially under different intervention scenarios.

In Great Britain (GB) there is a long running conflict between those seeking to conserve the legally protected hen harrier *Circus cyaneus* (L.) and those commercially shooting red grouse *Lagopus lagopus scoticus* (Lath.) (e.g., [22], [23]). Harriers and grouse breed on moorland dominated by heather *Calluna vulgaris* (L.) (Hull), and, under certain conditions, harriers can limit grouse populations and make commercial shooting uneconomic [24], [25]. As a result harriers are often illegally killed [26] and this limits their abundance and distribution across GB [27].

Although previous models of hen harrier distribution and potential population size exist (see, for example, [27]–[30]), these tend to be either statistical models based on spatial predictors from areas where hen harriers breed or mean-field models of temporal changes in harrier dynamics without spatial predictions of hen harrier distribution. An individual-based approach can simultaneously capture both the spatially-explicit nature of the problem (which the current statistical approaches seek to tackle) and the population dynamic processes (which the mean-field models represent) to explore spatial and temporal trends. This modelling approach can then be used to increase our understanding of the environmental and behavioural drivers of hen harrier population dynamics and also to facilitate *in silico* testing of potential harrier dynamics under different management scenarios.

In this paper we introduce an individual-based model for understanding and predicting the spatio-temporal dynamics of a hen harrier population. We applied this to habitat, prey and gamekeeper (grouse manager) management data from GB to simulate existing hen harrier population trends. The model was validated using empirical survey data. We examined the relative influence of several different model parameters on the population dynamics. Finally, we discuss potential future uses of the model in the context of conservation conflicts.

## Methods

As far as possible, the description of the model follows the ODD (Overview, Design concepts, Details) protocol [15], [31].

### 1. Overview

#### 1.1 Purpose

The model was designed to simulate the existing hen harrier population in GB. It was furthermore built to explore spatio-temporal population dynamics in the presence of spatial patterns in the level of grouse moor management to identify potential locations of conflict.

#### 1.2 Entities, state variables and scales

The model comprises four different entities: bird, cell, population and landscape. Birds and cells are low-level entities, the population comprising all of the birds present in the model at any one point in time. Likewise, the cells present in the model collectively form the landscape.

Individual birds simulate individual hen harriers and are characterised by a unique identity number, age, sex, breeding status, breeding success in the previous year, x and y coordinates of the natal cell and x and y coordinates of the cell the bird is located in (Table 1).

**Table 1:** Variables for the bird, cell, population and landscape entities in the individual-based hen harrier model.

Entity	Variable	Description (Unit)
Bird	ID	Identifier number, unique for each bird
	Age	Time since birth (years)
	Sex	Sex of the bird
	Status	Breeding status of the individual: 0: juvenile;1: non-breeder; 2: male: potential breeder; female:dispersed but will not breed this year; 3: breeder;4: failed breeder; … other values for dead birds …
	Success	Breeding success in the previous year
	Natal x	x-coordinate of the natal cell (km)
	Natal y	y-coordinate of the natal cell (km)
	x	x-coordinate of the current cell (km)
	y	y-coordinate of the current cell (km)
Cell	MP Index	Index of meadow pipit abundance
	Burning	Index of heather burning, proxyfor gamekeeper activity
	Suitability	Habitat-based index of suitability
	Heath	Combined area of open and dwarf shrub heath
	Resident Male	Pointer to the male bird set as resident of the cell
	Resident Female	Pointer to the female bird set as resident of the cell
Population	Adults	Vector storing pointers to Birds of age 1 or greater
	Juvs	Vector storing pointers to Birds of age less than 1
Landscape	Squares	Two-dimensional array, XMAX by YMAX,storing pointers to Cells

Cells represent squares of 1 km by 1 km on the ground. They are characterised by: meadow pipit, *Anthus pratensis* (L.), (MP) abundance, heather burning, habitat-based suitability (see [27] for more details), heath availability, mean altitude, the male bird resident in the cell and the female bird resident in the cell (Table 1). The first five characteristics are indices based on real data (see section 3.2, *Input data*, for more details). For the first stage of dispersal, an additional entity level exists, when cells around the location of the dispersing individual are grouped into landscape-scale (LS) squares. These are squares containing *n* by *n* cells, where *n* is given by the “Dimension of landscape-scale square” (LOCDIM; Table 2).

**Table 2:** Parameters and their default values in the individual-based hen harrier model.

Parameter	Description	Unit	Value	Source
MAXALT	Maximum suitable mean altitude	m	600	[32]
MINHEATH	Minimum area of heath in cellin order to be suitable	ha	20.0	[40]
LANDDIM	Dimension of landscape-scale search	Landscape-scalesquares	11	a
LOCDIM	Dimension of landscape-scale square	km	9	a
Distance-weightingparameters in dispersal				
KDISTNM	Post-natal, males	N/A	0.04	a
KDISTNF	Post-natal, females	N/A	0.50	a
KDISTFM	Post-failure, males	N/A	0.50	b
KDISTFF	Post-failure, females	N/A	0.90	b
Long-distance dispersal (LDD)of first-year Birds				
PLDD	Percentage making LDD movement	%	5.00	c
LDDMEANM	Mean distance, males	km	150.0	c
LDDSDM	Standard deviation in distance, males	km	67.0	c
LDDMEANF	Mean distance, females	km	100.0	c
LDDSDF	Standard deviation in distance, females	km	42.0	c
Breeding in Birds				
PBREED1M	Percentage of first-year males breeding	%	13.0	[26]
PBREED1F	Percentage of first-year females breeding	%	80.0	[26] d
PFAILGMOOR	Probability of complete nestfailure on grouse moor	%	63	[26] e
PFAILOTHER	Probability of complete nest failureon other habitat (i.e. elsewhere)	%	40	[26]
MFLEDGEGMOOR	Mean number of fledglings perfemale on grouse moor	N/A	4.26	[26]
MFLEDGEOTHER	Mean number of fledglings perfemale on other habitat	N/A	3.99	[26]
GMBIT	Grouse moor burning index threshold	N/A	2	
Winter mortality				
AMmort	Adult male	%	22	f
AFmort	Adult female	%	22	[26] g
JMmort	Juvenile male	%	64	f
JFmort	Juvenile female	%	64	[26] h
Persecution mortality^i^				
PERSmort0	On heather burning index of 0	%	0	j
PERSmort1	On heather burning index of 1	%	20	j
PERSmort2	On heather burning index of 2	%	40	j
PERSmort3	On heather burning index of 3	%	45	j
PERSmort4	On heather burning index of 4	%	50	j
PMF	Multiplication factor for persecution mortalities	N/A	1.0	

a The search area dimensions are based on expert opinion and chosen (along with the natal KDIST parameter values) to produce dispersal kernels matching, as closely as possible, reported natal dispersal distances (a data set partially overlapping with the raw data for [26] Table 11); see section 2.5 of Methods (but also see [37]).

b These were based on expert judgement and set to reflect the fact that hen harriers tend to disperse less far after breeding failure than from the natal location, and that males tend to disperse further than females.

c These values were set to allow for the rare extremely long distance dispersers as recorded in ([26] Table 11).

d PBREED1F was set to be higher than values in ([26] Table 10; 68%) because in the model some females that should breed fail to find a mate.

e The value of 80% ([26] Table 8) was adjusted to account for the fact that we model killing of nesting females separately.

f Assumed to be equal to the value for females.

g Table 14 (other moorland).

h p. 1092.

i Additional annual female mortality due to persecution.

j [26] p. 1098 suggested that additional mortality of females nesting on grouse moors is approximately 60%; our estimates are slightly conservative and scaled according to the level of heather burning observed (see section 3.2 of Methods (*Input data*)).

The population comprises the set of all the individual birds in the model and is characterised by two vectors, storing the characteristics of all adult and juvenile birds, respectively, in the model.

The landscape is the set of all individual cells in the model and is characterised by the spatial layout and individual properties of the cells. The model is spatially explicit, and the cells form a rectangular two-dimensional grid of 1205 by 599 cells, representing an actual area (between northings 12000 and 1217000, and eastings 55000 and 654000, of the Great Britain National Grid) which includes most of GB and associated islands (except the Isle of Man). One time step in the model represents one year.

#### 1.3 Process overview and scheduling

**Figure 1:**
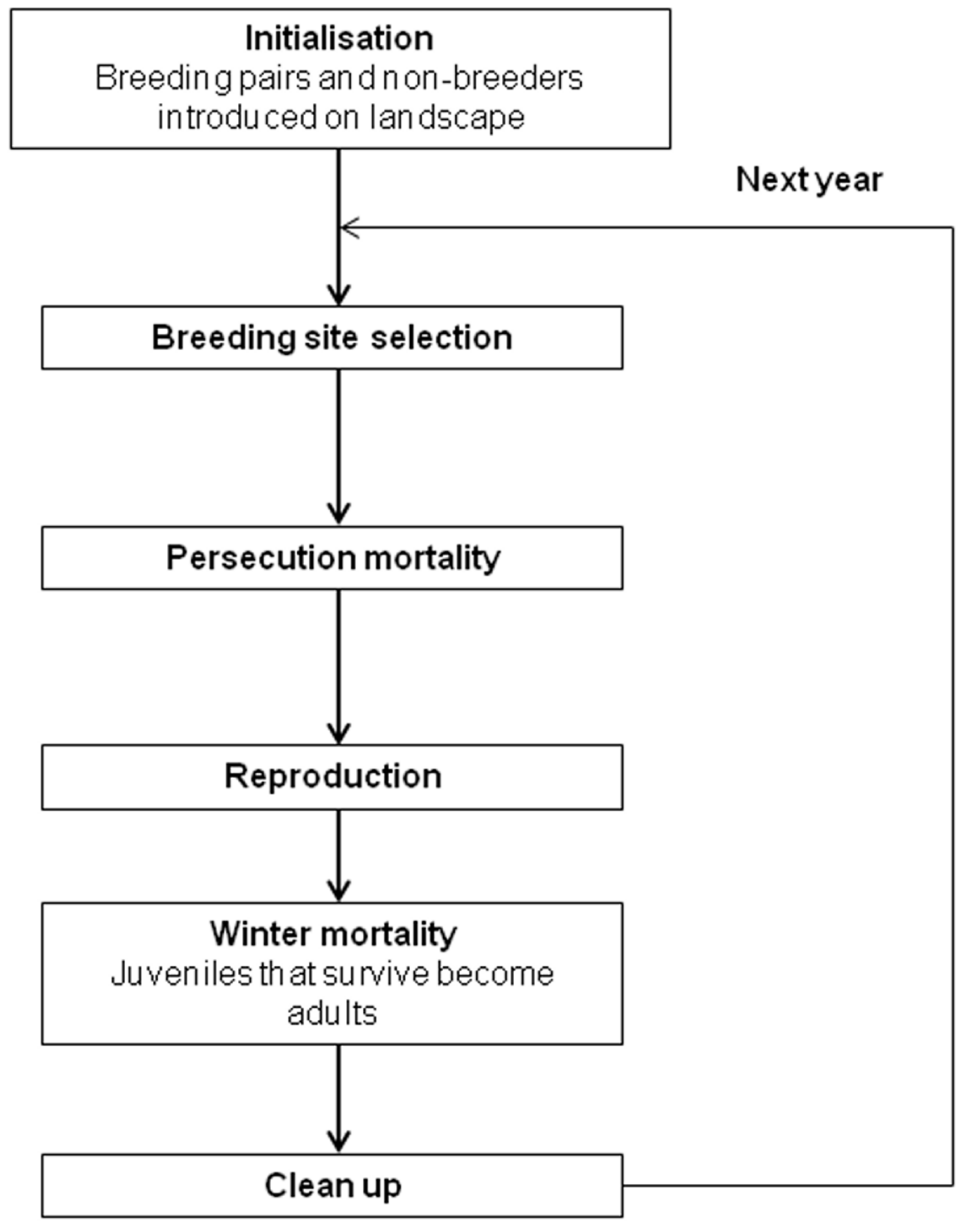
Flow diagram of model year cycle. A figure illustrating the main demographic and model processes in the annual cycle of the model.

The cells (apart from identities of residents) and hence the landscape are static, and are not changed after initialisation. For the birds, four main functions are performed in a typical year: breeding site selection, persecution mortality, reproduction and winter mortality (Figure 1). The year is simulated to begin in spring. Clean up, where dead birds are removed from the population, occurs at the end of each year (Figure 1).

Within each sub-model, adult and juvenile birds are processed separately and, in the first year, in the order they were added to the population. The order of adult birds is randomly shuffled at the end of each year. For more details, see the sub-models section (3.3).

### 2. Design concepts

#### 2.1 Basic principles

The model is built on the concept of individual-based modelling where system dynamics emerge from the behaviour of individual entities. The model is spatially explicit, running in discrete space and time.

#### 2.2 Emergence

Population dynamics and density distribution emerge from the behaviour of individual birds, which, together with the life cycle of individuals, are entirely represented by empirical rules.

#### 2.3 Adaptation

The dispersal behaviour of individuals is based on the environment and on their own status (see “Breeding site selection” in Section 3.3. for more details). Dispersing males are guided by indicators of real breeding site suitability based on theory and empirical observations. Dispersing females are guided by the presence of available territorial males. The rules by which individuals aim to select the most suitable habitat are fixed.

#### 2.4 Objectives

Individuals are modelled implicitly to aim to maximise the suitability of their nesting site but with imperfect decision-making, and productivity is not a function of this suitability. Individuals that breed successfully will stay in their cell and with their partner, but they have no option to modify this behaviour to maximise their objective. Individuals that do disperse can seek to maximise this objective under relatively tight restrictions. Males seek to find the best breeding site, which is ranked according to the index of meadow pipit abundance of the cell (for the LS square selection, this is in relation to the number of resident male hen harriers already present in the square), as a proxy of prey availability, with thresholds of heath availability, habitat-based suitability and mean altitude as controls on habitat suitability for nesting, and distance from the current location as a measure of dispersal cost (only for the LS square selection). Females evaluate the desirability of an LS square on the basis of the number of resident males without females present in the site and the distance from the current location. A cell with an available resident male is chosen within the LS square based on the same heath, habitat and mean altitude restrictions and meadow pipit abundance scores as used for males selecting a cell within an LS square.

#### 2.5 Sensing

Individual birds are assumed to know the prey, heath and suitability index values and mean altitude of the cells surrounding their location (the bird is at the centre of the search area, the dimensions of which, in LS squares, are given by LANDDIM; Table 2) in any one year, the distances to each cell and whether there are resident males and females in each. The process by which birds are modelled to obtain this information was developed based on some of the authors’ knowledge and following discussions with raptor experts. See “Breeding site selection” in the *Sub-models* section for more details.

#### 2.6 Interaction

Interactions between individual birds are indirect, through competition for cells between males and for males between females.

#### 2.7 Stochasticity

When non-juvenile individuals are introduced to the population, the age of each individual is randomly sampled from the range specified. All mortality events include an element of stochasticity; the individuals which die are therefore randomly chosen based on the mortality probability as defined by the mortality parameters (Table 2). Likewise, first-year birds which perform a long-distance dispersal event (see “Breeding site selection” in section 3.3) are chosen at random from all the first-year birds in the population. First-year males and females which become breeders are chosen at random from all first-year males or females in the population, respectively. Random choices are not made by selecting a fixed number of individuals but by applying the probability of the event occurring to each individual in the list in turn. The number of offspring is sampled from a Poisson distribution with a mean corresponding to the appropriate parameter, for each successfully breeding female. Dispersal is probabilistic, with cells or LS squares with a higher score having a higher probability of being chosen as the dispersal destination.

#### 2.8 Observation

In order to validate the model using empirical observations, the total numbers of breeding females (and hence potentially breeding pairs) in the model at the stage between “Breeding site selection” and “Persecution mortality” are recorded from the model in each year. At the same time, the number of breeding females in cells classified as heavily managed grouse moorland (having a burning index value greater than GMBIT; Table 2) and those in other cells are recorded, together with regional numbers (see section 3.1, *Initialisation*, for more details about regions). Matrices showing the spatial location of each potential breeding pair (here termed “breeding pairs”) are recorded for specified years and numbers are averaged for each cell across all replicates. After all breeding pairs have bred, the numbers of successful pairs and the total number of fledglings are recorded, each separately for managed grouse moorland and other habitat.

### 3. Details

#### 3.1 Initialisation

**Figure 2:**
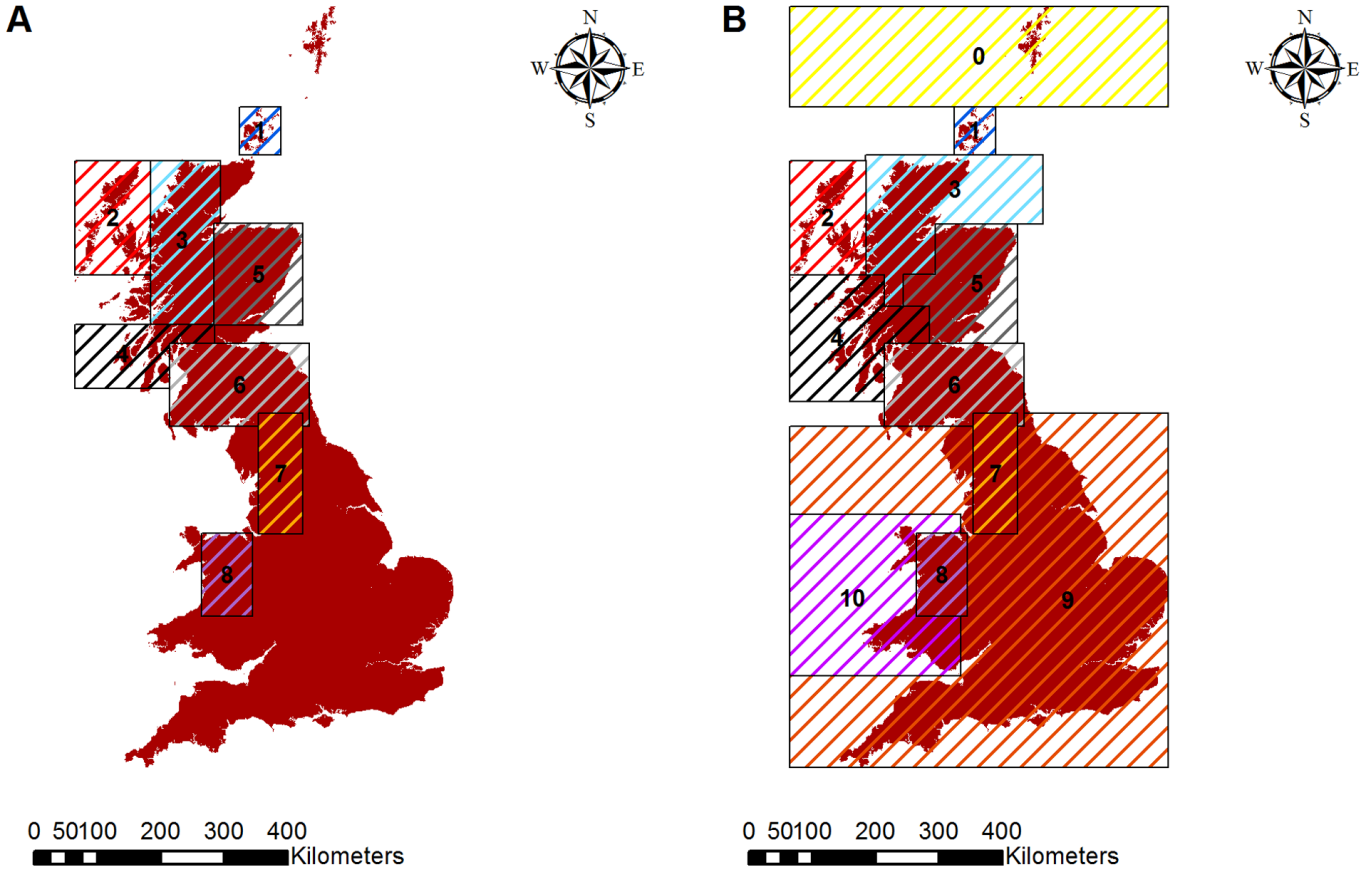
Regional boundaries in the model. (A) Regional boundaries in the initialisation of the hen harrier population; (B) Regional boundaries used in counting numbers of breeding pairs. The numbers and colours correspond to region names as follows: 0– Shetland; 1– Orkney; 2– Hebrides; 3– North Highlands; 4– West Highlands; 5– East Highlands; 6– Southern Uplands; 7– England (census breeding pair counts only); 8– Wales (census breeding pair counts only); 9– All of England (includes area of ‘7’); 10– All of Wales (includes area of ‘8’).

The landscape is created from input data at the beginning of the scenario. The initialisation of the population and birds depends largely on the scenarios being investigated. A specified number of male-female pairs are “released” into random cells within rectangular regions bounded by user-supplied co-ordinates (Figure 2A). These are slightly more simplistic than the boundaries used to define regions for the purposes of output (Figure 2B). Only one pair can be released into any one cell. Adults released are set as the resident breeders of the square and the year will begin with the site selection and dispersal of the adults, which is the commencement of the breeding season. Ages of individuals are set at random between two and five years inclusive, and all individuals are assumed to have bred successfully the previous summer.

The initial setup is set to resemble the size and spatial distribution of the hen harrier population according to the 1988–1989 census (see [32] Table 3): 71 adult pairs are released in Orkney, 20 in the Hebrides, 85 in North Highlands, 148 in West Highlands, 109 in East Highlands, 80 in Southern Uplands, 18 in England and 27 in Wales. In addition to breeding pairs, a number of non-breeding male and female adults are released into each region at the start to account for the non-breeding component of the hen harrier population. The number of non-breeders released was set as 42% of the number of breeding pairs. This percentage was obtained from the ratio of non-breeding males to breeding pairs in preliminary simulation runs where no non-breeders were introduced, in the time period 1989–2030 and after the ratio reached a relatively stable value. Non-breeders were released in male-female pairs but not assigned as residents of cells. All non-breeders were initialised as one-year-olds.

**Table 3:** Breeding success and fledgling numbers from hen harrier simulations.

	Baseline scenario (SE)^a^		Census estimates (SE)^b^	
	Grouse moor	Other habitat	Grouse moor	Other habitat
Proportion attempts successful	0.203 (0.011)	0.573 (0.010)	0.197 (0.023)	0.599 (0.032)
Fledglings female^−1^ year^−1^	0.867 (0.055)	2.295 (0.045)	0.840 (0.123)	2.390 (0.191)

a Baseline scenario refers to the simulations run where MAXALT = 600 m, MINHEATH = 20.0 ha, PMF = 1.0, JMmort = 64%, GMBIT = 2 and all other parameter values are set at their default values as described in Table 2. Breeding parameter estimates were calculated across all females attempting to breed, across all years from 1989 to 2030 and across all replicates.

b Census estimates come from ([26] Table 8).

#### 3.2 Input data

The model utilises five spatially referenced datasets in the form of raster data layers, each having a grain size of 1 km:

***Meadow pipit abundance index.*** The meadow pipit index is appropriate for describing prey availability for hen harrier breeding in heather moorland. Three different data layers, based on different models of the same data, were trialled in the model as the input data for prey availability. These were two-stage models (applied to handle substantial over-dispersion in MP annual counts due to absence from many sampled squares), comprising a generalised linear model (GLM) assuming a binomial error structure for predicting presence and a second model to predict abundance in squares where presence is predicted, using one of the following: (i) a GLM with log link function and Poisson error term, (ii) as (i) but adjusted for under-prediction and (iii) a generalised additive model (GAM) with identity link function and normal error term. These were based on data from the Breeding Birds Survey of the British Trust for Ornithology, 1994–2007 ([33], [34], Text S1). The models predict the number of MPs seen per 2 km transect surveyed in each 1 km^2^. The GAM produced the highest correlation with the estimation data set and was close to the GLMs when correlated with the validation data set (Text S1). The GAM also avoided unrealistically high maximum abundances predicted by the adjusted GLM (43.5 vs. 119; Text S1) and it was therefore used for all cells for all simulation runs of the current hen harrier model.

***Heather burning index.*** The heather burning index for each cell was produced from the 10 km Ordnance Survey grid of the proportion of heather burnt in strips visible from aerial photographs [27]. This was done by assigning the value of each 10 km by 10 km square to every cell that fell within the range of its coordinates. This was used as an estimate of gamekeeper management activity [35], [27], and hence an indirect index of illegal hen harrier persecution, since an important component of red grouse management is heather burning. The burning index values were set as a discrete scale from 0 to 4 following pre-established categories ([27] Fig. 2f).

***Heath availability.*** The heath availability data are the combined areas of dense and open dwarf shrub heath in the cell, from the Land Cover Map 2000 (NERC Centre for Ecology and Hydrology).

***Suitability index.*** The suitability index values are the output of a habitat-based model of potential hen harrier distribution in the absence of persecution [27].

***Mean altitude.*** The altitude values are mean altitudes extracted for each square from the Countryside Information System [36].

#### 3.3 Submodels

***Breeding site selection.*** All males are processed first. Individuals which did not breed successfully in the previous year will disperse. Females which bred successfully in the previous year but whose mate has died during the winter will also disperse.

A proportion of first-year individuals perform a long-distance dispersal movement prior to searching for a suitable cell. The distance to move is sampled from a normal distribution with a sex-specific mean and standard deviation (Table 2), which are user-defined and remain constant for all individuals throughout the simulation. The direction of the movement is sampled randomly. If the destination cell is on land, the individual moves to that location, which becomes the centre of the site selection process. An individual can attempt to find a destination on land up to nine times. If all attempts fail, the individual begins the site selection process from its current location.

**Figure 3:**
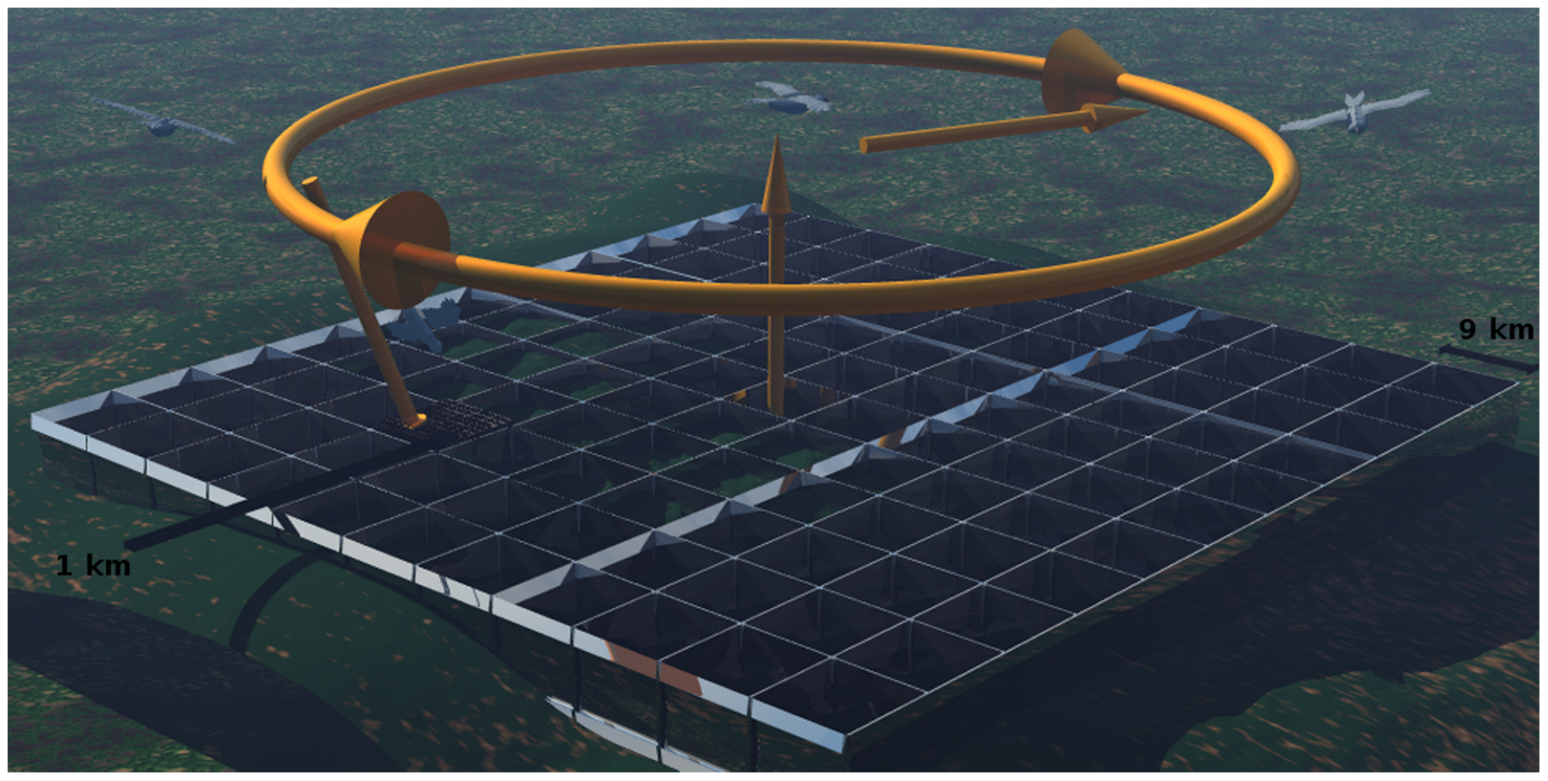
Illustration of modelled hen harrier breeding site selection behaviour. Selection of landscape-scale (LS) square (9 km by 9 km square) and selection of cell (1 km square) within selected LS square.

There are two stages in the process by which an individual selects a cell as a territory. This structure is designed to incorporate ecological realism into the simulations and was based on an approximation of expert opinion of the methods used by hen harriers when choosing a nesting site (Figure 3).

At the landscape-scale, the score of the LS square for a male is calculated using the total index value, being the weighted sum of the meadow pipit index values of the cells within the LS square. The area of heath for a single cell must be greater than or equal to the minimum area of heath (MINHEATH; Table 2) or else the score for the cell will be considered as zero. Furthermore, the suitability index value for the cell must be greater than or equal to 0.0000001 (effectively any value above zero) or the score of the cell will also be zero. The mean altitude of the cell must also be lower than or equal to the altitude threshold (MAXALT; Table 2) in order for the score to be above zero.

For males, the score for each LS square is also weighted according to the number of resident males in the LS square in relation to the variable *K*, calculated for each cell *i* in the LS square, which describes the carrying capacity of the LS square in terms of the abundance of food as described by the meadow pipit index (equation 1).
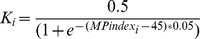
(1)


This is done in order to avoid unnaturally high densities of territorial pairs. The shape of *K_i_* was determined from visual examination of the relationships between hen harrier densities and the abundance of meadow pipits ([24] Figure 3), and the factor of 0.5 accounts for MP abundances in ([24] Figure 3) being per 1 km of survey transect rather than per 2 km as in the data used here in the model.

The score of the LS square is multiplied by the difference between the sum of the carrying capacities of the cells and the number of territorial hen harrier males in the LS square, and divided by the sum of the carrying capacities (equation 2).
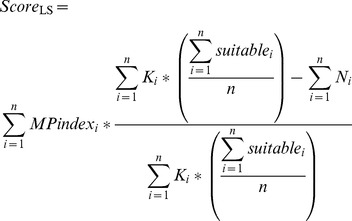
(2)
*N_i_* is the presence (1) or absence (0) of a territorial hen harrier male in cell *i* of the LS square, *K_i_* is the value of *K* of the individual cell *i* in the LS square and *n* is the number of cells in the LS square, given by LOCDIM*LOCDIM (Table 2). Where an LS square contains suitable cells, the sum of *K_i_* values is also multiplied by the proportion of cells in the LS square that are suitable, to adjust LS carrying capacity accordingly (equation 2). “Suitable” refers to cells which meet the threshold conditions described above. Where no cells in the LS square are suitable, the score is set to zero. This calculation of *K* allows cells in the square which may be available as foraging grounds to influence the decision of the hen harrier while restricting the carrying capacity based on the available nesting sites.

For females, the score of the LS square is equal to the number of cells within it which contain a resident male but which do not contain a resident female (we assume all individuals to be monogamous). All male dispersers will have dispersed before females make decisions on dispersal.

For all individuals, every LS square within the dispersal boundary (*i.e.* within the set of LS squares being searched, given by a square of *m* by *m* LS squares centred around the cell in which the individual is located, where *m* is the dimension of the LS search (LANDDIM; Table 2)) is then weighted by multiplying the score of the LS square by the distance weight (*Weight_d_*), an inverse exponential function of the distance (*d*) between the centre of the square and the location of the bird before dispersal, i.e. the centre of the search (equation 3).
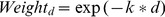
(3)Here, *k* is the appropriate distance-weighting parameter (Table 2) and *d* is the Euclidian distance, in numbers of cells, from the coordinates of the location of the individual to the centre of the LS square. The individual selects an LS square, with the probability of selecting a square being proportional to its score.

The individual then selects a cell as a territory (defined here as the core nesting and foraging area of the individual; 1 km^2^ in size – see [37]) within the selected LS square. The selection process is in most respects identical to the procedure used in selecting the LS square. Since a single cell is under consideration at any point in the search, there is no need to sum up scores. For males, cells which already have a male resident are given a score of zero. For females, squares must have a resident male and no resident female, and must not be the natal site of the female, in order to be allocated a non-zero score. There is no weighting of cells by distance or by *K*. For both males and females, cells which meet these criteria and the heath, suitability and altitude criteria are given scores proportional to their MP index values.

Specified proportions of first-year males and females are allocated as non-breeders (Table 2) to reflect the frequency distributions of age at first breeding as reported ([26] Table 10). Individuals which become potential breeders are allocated as residents of their cells. If a cell contains a female, and therefore a pair (since at this stage in the dispersal process females only move to a cell with at least one male present), the resident male and female are identified as breeders.

If all the cells in the LS square are totally unsuitable, i.e. have a score of zero, the individual is set as a non-breeder and remains in its original location. For a female, if all the landscape-scale squares in the search area have a value of zero, the individual moves at random by one LS square for a second attempt at site selection and dispersal. If the female fails to find a suitable cell on the second attempt it remains at the location where it started its new attempt and becomes a non-breeder.

***Persecution mortality.*** Persecution of hen harriers on heavily managed grouse moors is simulated by applying a habitat-specific probability of mortality, multiplied by a persecution mortality factor (PMF; Table 2) to all breeding female individuals (*i.e.* those with a status of “3”) following dispersal. The PMF allows variation of the overall level of persecution mortality without changing the relative mortalities with respect to other burning index values (Table 2). In baseline scenarios, cells with a burning index value of greater than two are considered as heavily managed grouse moorland (GMBIT = 2; Table 2). We assume that there is no significant persecution of breeding males and other adult birds. Chick mortality is incorporated into the probability of complete nest failure in Reproduction (see below).

***Reproduction.*** Breeding females of age one or more will reproduce. A probability of nest failure (Table 2) is applied to the female. For failed females, the number of fledglings is recorded as zero. Otherwise, the number of fledglings is drawn from a Poisson distribution with a specified mean, given by the corresponding parameter for the burning index value of the cell (GMOOR if burning index>GMBIT; otherwise OTHER; Table 2) and based on mean successful brood sizes from data presented in ([26] Table 7). For the parameter values, the empirical values were multiplied by the average number of breeding attempts (for grouse moor and other moorland, respectively), to account for multiple breeding attempts. If the resultant number of fledglings for a breeding female is zero, this is set as one, since the nest has already been determined as successful. Pairs with fledglings are recorded as successful, while those with no fledglings are set to have a status of “failed breeders”. The fledglings are initialised as juveniles of age zero in the territory of their parents, each with a probability of 0.5 of being male.

***Winter mortality.*** Mortality is not sex-specific by default, but differs between adults and juveniles. Individuals which die have their status changed but are not yet removed from the population. Juveniles which survive are recorded as adults, and the juvenile vector is emptied for the coming year.

***Clean up.*** Adult birds which have died during the year are removed from the population (no individuals perform any of the behaviours described after they are classed as dead, even though they remain in the vector until this stage). If a dead adult is a resident of the cell it is located in, the cell is updated as having no resident of the sex of the dead adult. The order of adult birds in the list is shuffled into a random order.

### 4. Simulation experiments

We ran two different sets of simulations: (i) model validation and (ii) elasticity analyses.

#### 4.1 Model validation

The number and distribution of pairs of birds as initialised was set to mimic the regional numbers of breeding pairs recorded in Scotland, England and Wales in the 1988–89 survey ([32] Tables 2 & 3). Regional numbers of breeding pairs (does not include non-breeders or juveniles) in the 10^th^, 16^th^ and 22^nd^ years of the model simulations were compared to the results from the 1998, 2004 and 2010 surveys [32], [38], respectively (the first year was considered to be 1989). The mean numbers of fledglings per breeding pair and the proportion of successful pairs were calculated separately for heavily managed grouse moorland and other habitat from the output data. Breeding pairs included those where the female was killed due to persecution. The results were compared to empirical data in ([26] Table 8) to validate the model parameter values. The scenario was run for 50 years in order to examine short-term population dynamics beyond the census years, and replicated 20 times.

#### 4.2 Elasticity analyses

We then assessed the sensitivity of the model to five parameters: the threshold for the maximum mean altitude of a cell in order for it to be suitable as a territory for a hen harrier (MAXALT), the threshold for the minimum area of heath required in a cell for it to be suitable as a territory for a hen harrier (MINHEATH), the proportion by which the original persecution mortality values were multiplied to vary the levels of female persecution mortality but keeping them the same relative to each other (PMF), the juvenile male winter mortality (JMmort; Table 2) and the threshold value of heather burning above which habitat is classified as managed grouse moor (GMBIT). These parameters were chosen to represent straightforward environmental variables as well as key mortality parameters. Although few hen harrier nests in Great Britain have been found above 600 m (see [32]), the possibility of excluding potential nest sites or including inappropriate nest sites (if the threshold is too low) was regarded as worth investigating. We examined sensitivity to both of the additional threshold parameters we set for nest site selection; altitude and heather cover. Heather is the preferred habitat of hen harriers in Britain [39] and although its presence might not improve breeding success it may affect the site selection and hence distribution of hen harriers in Great Britain [40]. Persecution is a fundamental issue of the conservation conflict surrounding hen harriers and hence the sensitivity of the breeding hen harrier population to the level of persecution is of high importance. Juvenile hen harrier winter mortality estimates are low [26], [41], and it may be an important demographic factor in population growth in hen harriers. For female juveniles there are relatively good estimates of winter mortality (see [26] p. 1092) but for males estimates are less certain [26]. Finally, since persecution is related to gamekeeper activity on grouse moors, we investigated whether the criterion for distinguishing between (heavily) managed grouse moor and other habitat would have a significant impact on population dynamics.

Only one parameter was varied at any one time. The value of the parameter under investigation (*P*) was varied by 10% (*P/*10) either side of the baseline value, and the elasticity was calculated as the ratio of the proportional change in the number of breeding pairs (*BP*) to the proportional change in the parameter value (equation 4).
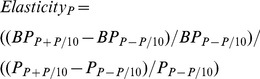
(4)Where the parameter value had to be an integer, the altered parameter value (‘minus 10%’ or ‘plus 10%’) was rounded to the nearest integer and the difference in parameter value (equation 4) was adjusted accordingly. It should be noted that because the changes in *BP* and *P* are divided by values at the lower end of the parameter space (*P*-*P*/10) rather than the baseline value itself (*P*), the elasticity values will differ slightly from the elasticity values around the baseline parameter value.

Elasticities were also calculated for the regional numbers of breeding pairs and numbers of breeding pairs nesting in heavily managed grouse moor and those nesting in other habitat, and the numbers of fledglings per breeding pair. For each set of elasticities, the corresponding regional number of breeding pairs or the mean number of fledglings per breeding pair was used instead of the national total of breeding pairs, as appropriate.

We also examined the interaction between two survival parameters with respect to change in the numbers of breeding pairs nationally. We ran a set of 35 parameter scenarios, for each combination of the PMF values 0.0, 0.5, 1.0, 1.5, and 2.0 and the juvenile (male and female) winter mortality values 0, 16, 32, 48, 64, 80 and 96%. For each scenario, 20 replicates were run.

For all scenarios where they were not varied, MAXALT was set as 600 m, MINHEATH was set as 20.0 ha, PMF was set as 1.0, JMmort was set as 64% and GMBIT was set as 2. Each scenario was run for 50 years and replicated 20 times.

### 5. Analysis of model output

The number of breeding pairs was calculated after breeding site selection and before persecution mortality to include females which in real life would have attempted to breed but would have been killed through persecution. The number of breeding pairs was calculated as the number of females which met the breeding conditions (aged 1 year or more, the resident female in the cell and with a resident male present in the same cell) at the end of the breeding site selection process. Total numbers of breeding pairs across the entire model landscape were compared to the UK totals estimated by ([32] Table 2, [38] Table 3) less the totals for Northern Ireland. Regional totals were compared to respective estimates ([32] Tables 2 & 3, [38] Tables 3 & 4).

**Table 4:** Elasticity of the simulated model output in selected years to five model parameters, in grouse moor, other habitat and overall.

Model output	Region	Year	Parameter				
			Altitude thresholdMAXALT (m)	Heath thresholdMINHEATH (ha)	Persecution mortalityfactor PMF	Juvenile male mortalityJMmort (%)	Grouse moor burning indexthreshold GMBIT
Mean numbers ofbreeding pairs	Great Britain	1998	−0.063	−0.281	−0.488	−1.525	0.044
		2004	−0.023	−0.222	−0.510	−2.653	0.050
		2010	0.174	−0.254	−0.480	−3.318	0.065
		2030	0.425	−0.278	−0.284	−3.587	0.041
	Grouse moor	1998	0.022	−0.031	−1.682	0.045	−0.410
		2004	−0.200	0.129	−1.268	−1.923	−0.410
		2010	−0.109	−0.489	−1.186	−2.893	−0.411
		2030	0.557	−0.401	−0.895	−3.996	−0.379
	Other habitat	1998	−0.075	−0.312	−0.301	−1.688	0.140
		2004	−0.005	−0.255	−0.428	−2.719	0.123
		2010	0.198	−0.233	−0.409	−3.353	0.129
		2030	0.412	−0.265	−0.215	−3.534	0.106
Mean numbers of fledglingsper breeding pair^a^	Great Britain	1998	−0.063	0.011	−0.024	−0.177	0.016
		2004	0.095	−0.123	0.031	−0.159	0.005
		2010	0.055	0.066	0.105	−0.201	0.003
		2030	−0.055	0.006	0.011	0.151	0.009

a Only for the overall population.

The mean numbers of fledglings per breeding pair in heavily-managed grouse moorland and in other habitat were calculated separately by averaging the total numbers of fledglings produced by all females in cells of the respective habitat type across the total number of breeding pairs in the same habitat type in that year. The means were then averaged across all replicates and all years from 1989 to 2030. The proportions of successful breeding pairs were calculated similarly, but using total numbers of females producing at least one fledgling instead of total numbers of fledglings.

For elasticity scenarios, changes in numbers of breeding pairs (national and regional) and mean numbers of fledglings with parameter values were only assessed for the years where census data were available (1998, 2004 and 2010), and for the last year of a short period of extrapolation (2030).

## Results

### 1. Model validation

**Figure 4:**
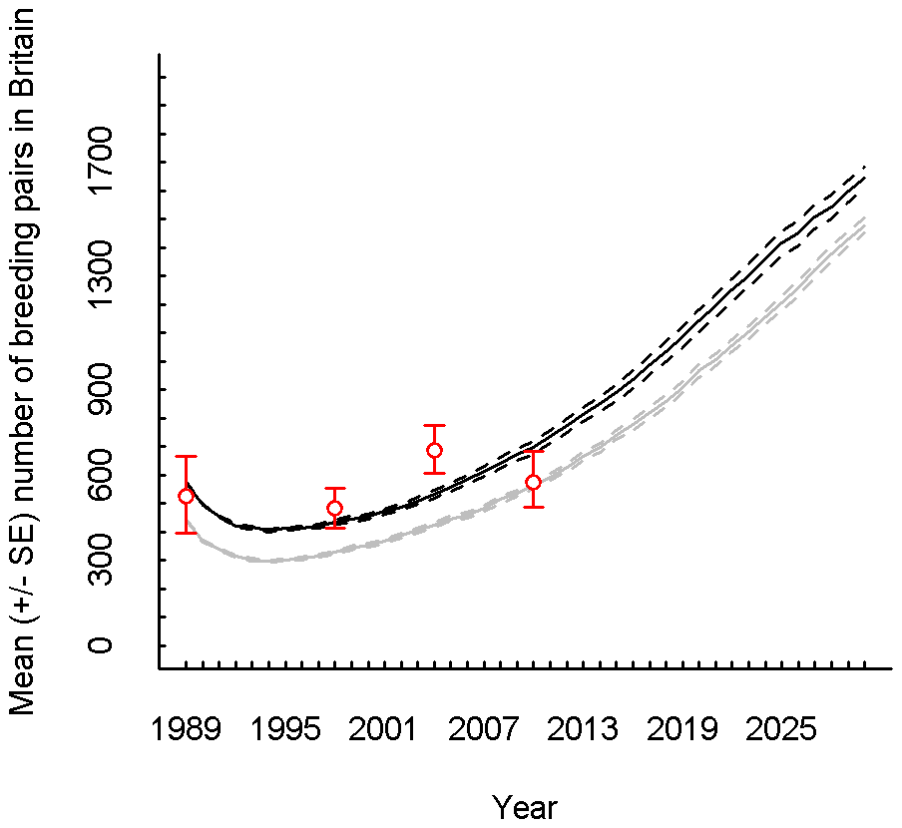
Simulated national hen harrier breeding population growth between 1989 and 2030. The mean (± standard error) of the annual number of breeding pairs of hen harriers averaged across twenty replicate simulations. “Breeding pair” refers to a male and a female in a single territory which attempt to breed, whether successful or not. Black: non-breeding males and females of age 1 were added in the initialisation (SE range: 0.8–47.8). Grey: no non-breeding males or females introduced at the start (SE range: 2.1–27.5). Dashed lines indicate SE boundaries. Red circles denote the estimates and the red error bars indicate the range of the 95% confidence intervals from the censuses [32], [38]. MAXALT = 600 m, MINHEATH = 20.0 ha, PMF = 1.0, JMmort = 64% and GMBIT = 2.

For the baseline scenario (MAXALT = 600 m, MINHEATH = 20.0 ha, PMF = 1.0, JMmort = 64%, GMBIT = 2), the breeding population began with an average of 576 pairs (Figure 4). Thus the actual breeding population was on average 52 pairs greater than the estimated national population size (524 pairs; UK total less Northern Ireland; [32]). The initial breeding population size set for the model was higher than the census estimates, at a total of 558 pairs (broken down by region in section 3.1 of the methods; *Initialisation*); this was due to the difference between the sum of the regional census estimates and the total national census estimate ([32]; for the reasons behind this, see the legend of Table 3 in [32]). No mortality events had occurred by this stage in the model. Therefore, on average, 18 pairs across the model landscape must have changed status from non-breeders to breeders during the site selection process in the first year of the model run.

The model breeding population declined sharply between counts in the first year and the second year, and continued to decline, albeit at a decreasing rate, until the sixth year of the model run (1994, Figure 4). Beyond this, the national population increased at a slowly increasing rate, reaching 435 pairs in the 10^th^ year of simulation (1998, Figure 4). The average figure was 48 pairs lower than the census estimate for 1998 (483 pairs; [32] Table 2). By 2004 the gap between the census estimate and the model result had widened, with 533 pairs in the model compared to an average estimate of 686 for the UK minus Northern Ireland ([32] Table 2). However, the model simulated higher numbers of breeding pairs for 2010 than the census estimate (696 *vs.* 574). In all years except 2004, model results overlapped at least partially with 95% confidence intervals for the census estimates (Figure 4). Beyond census estimates, the population growth appeared relatively linear, with some indication of a reduction in growth rate near 2030 (Figure 4). The addition of non-breeders at the start of the model run raised the national breeding population size for each year considerably, and produced a better overall match to census estimates (Figure 4).

**Figure 5:**
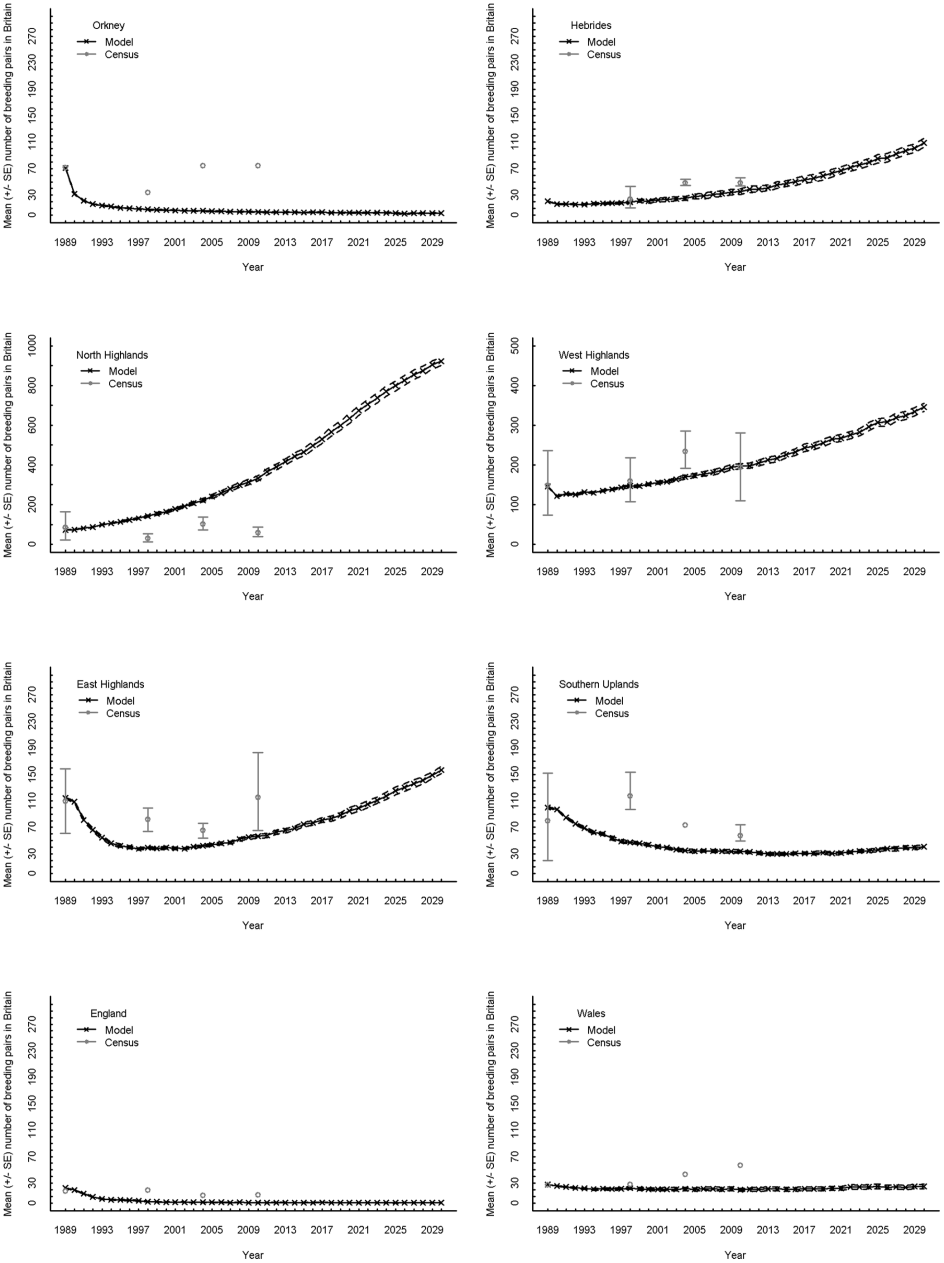
Simulated regional breeding population growth between 1989 and 2030. The mean (± standard error) of the annual number of breeding pairs of hen harriers averaged across twenty replicate simulations. “Breeding pair” refers to a male and a female in a single territory which attempt to breed, whether successful or not. Crosses joined with lines denote mean values while dashed lines indicate SE boundaries. Grey circles denote the estimates and the grey error bars indicate the range of the 95% confidence intervals from the censuses [32], [38]. MAXALT = 600 m, MINHEATH = 20.0 ha, PMF = 1.0, JMmort = 64% and GMBIT = 2. Note the different y-axis scales for North and West Highlands. For England and Wales, model results are presented for cells within the limits of the 2004 census only ([32] Figure 2).

**Figure 6:**
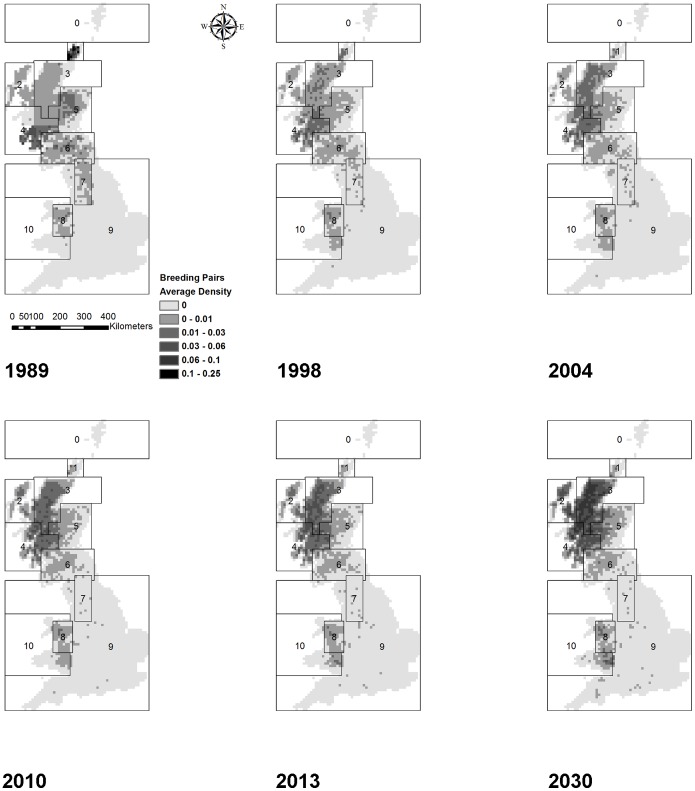
Spatial patterns in hen harrier population growth, 1989–2030. Average densities of simulated pairs attempting to breed in each 10 km by 10 km square on the landscape for the baseline scenario, for the years 1989, 1998, 2004, 2010, 2013 and 2030. Average density is calculated as the average occupancy of a cell across 20 replicate simulations, averaged across all 100 cells in the square. For example, an average density of 0.01 indicates that, on average, 1 out of 100 cells are occupied in the given year in any given replicate of the scenario. Black lines denote region boundaries. MAXALT = 600 m, MINHEATH = 20.0 ha, PMF = 1.0, JMmort = 64% and GMBIT = 2. For region names corresponding to numbers, see legend of Figure 2.

The regional breakdown of the population trend revealed that most of the breeding population settled in North Highlands (Figures 5–6); this was in contrast to census results where the highest estimates were for West Highlands ([32] Table 3, [38] Table 4). Other regional trends varied with respect to how well they reflected census estimates (Figure 5, [32] Tables 2 & 3, [38] Tables 3 & 4). For Orkney, the model subpopulation showed a dramatic decline rather the temporary decline and subsequent return seen in census estimates. For both Southern Uplands and East Highlands, the model predicted a decline followed by a subsequent increase, with the Southern Uplands increase beginning much later, after years of a low, relatively steady subpopulation.

For England and Wales, model subpopulation sizes which took into account only the census squares were compared to census estimates, but the total subpopulation sizes were also examined. Both England subpopulation estimates showed a rapid decline to almost no breeding pairs within the period of the census years (Figures 5, 6). The empirical estimates also indicated a decline, but more gradual, with 11 pairs remaining in 2004 and 12 in 2010 (Figure 5, [38] Table 3). For the census square areas of Wales, the subpopulation was predicted by the model to remain relatively constant throughout the census years and until 2030, but an increase was predicted across the whole of Wales (Figures 5, 6). The empirical census estimates for Wales showed a greater increase between 1998, 2004 and 2010 ([32] Table 2; [38] Table 3).

Visual inspection of the spatio-temporal patterns in numbers of breeding pairs indicated a retraction of the subpopulation in Scotland and northern England towards the North Highlands between 1989 and 2013, and possibly continuing in subsequent years, with an expansion back into some of the previously occupied areas – particularly in the Hebrides and East Highlands – by 2030 (Figure 6). Breeding pairs all but disappeared from England in the first 11 years (Figure 5), and the subsequent subtle growth seemed to be mainly due to an expansion of the Welsh subpopulation into the English region (Figure 6). The Welsh subpopulation dispersed from the northern part of Wales to the south, although the area in between the two subpopulations remained sparsely populated (Figure 6).

Proportions of successfully breeding pairs from the model corresponded well to reported proportions (Table 3, [26] Table 8). The probability of success was much higher outside habitat classed as (heavily managed) grouse moor (burning index greater than two) than within this habitat. Likewise, the mean number of fledglings per year was much higher for habitat other than grouse moor, model values being close to estimates from empirical data (Table 3, [26] Table 8). Values from the model were slightly different when calculated only across the years 1989–1995 to mimic the range used for the empirical estimates ([26] Table 8). However, even for 1989–1995 all mean values except the proportion of breeders on habitat other than grouse moor that were successful were within standard error limits of ([26] Table 8), and even this value was only outside the margin by a value of 0.011 (results not shown). Proportions of successfully breeding pairs and mean numbers of fledglings showed relatively minor trends, with both estimates for ‘other habitat’ (other than grouse moor) increasing in the first 20 or so years of the model run before largely stabilising, with an indication of a slight decrease until the end of the simulated years. On grouse moor, both estimates showed relatively high variation throughout but no clear trend with time.

### 2. Elasticity analyses

The elasticity analyses showed that, for all the different response variables utilised, JMmort had almost invariably the greatest proportional effect on the response variable (Tables 4, S1). The elasticity analyses for the MAXALT parameter showed that the relative effect of the altitude threshold on the national breeding population size increased dramatically with years, from a 0.63% change in the breeding population with a 10% change in MAXALT in 1998 to a 4.25% change with the same parameter value change in 2030 (Table 4). The magnitude of the influence of MAXALT on breeding pair numbers did not seem to vary dramatically with region, with the exception of small subpopulations such as England (census squares only; Table S1). There was no clear distinction in the influence of MAXALT between grouse moor and other habitat, with elasticity values varying rather widely between years (Table 4). Effects on mean numbers of fledglings per breeding pair were in the range of −1% to 1% change with a 10% change in MAXALT (Table 4).

The influence of the heath threshold (MINHEATH) on the national breeding population size was considerably steadier over years (Table 4). Elasticities for the national breeding population size ranged from −0.222 (2.22% change in population size for a 10% change in MINHEATH) to −0.281, with increase in MINHEATH always having a negative effect on population size (Table 4). Comparing habitat types revealed that elasticity to MINHEATH was more subtle in grouse moor in the early years, but higher in the later years (Table 4). The mean numbers of fledglings were most significantly affected by the change in MINHEATH in 2004; the influence being relatively low otherwise, especially in 1998 and 2030 (Table 4).

The influence of PMF on the national breeding population size was relatively high compared to the influence of both the altitude and heath thresholds (Table 4). The negative influence of increased PMF seemed to lessen in the later years (2030; Table 4). Regionally, the effect was also almost always negative (Table S1). Regions with relatively high densities of managed grouse moorland (East Highlands, Southern Uplands, England and, to a lesser extent, Wales) had relatively high elasticity values for persecution mortality (Table S1). As could be expected, elasticities were considerably higher for grouse moor numbers than for those nesting in other habitat (Table 4). Both sets of breeding pairs showed a general decrease in the influence of PMF on numbers over the years (Table 4). The influence on the mean number of fledglings per pair was more subtle and actually positive in the later years (Table 4).

Juvenile male winter mortality (JMmort) had the greatest proportional influence on the national breeding population size out of the five parameters examined (Table 4). The influence was always negative and increased with time, the population size varying by as much as 36% by 2030 for a 10% change in JMmort (Table 4). At the regional level, in most cases JMmort also had a greater influence on breeding subpopulation size than the three parameters already considered (MAXALT, MINHEATH and PMF) (Table S1). For both heavily managed grouse moor and other habitat, the proportional influence of JMmort increased with time (Table 4). It is worth noting that the influence of JMmort was rather small and actually positive for grouse moor in 1998, although effects were negative in all other cases, for both habitat types, and exceeded the influences of the other parameters investigated (Table 4). The influence of JMmort on the mean numbers of fledglings per pair, although generally much lower than the effect on population sizes, was still considerably greater than the influences of other parameters investigated and mostly negative (Table 4).

The grouse moor burning index threshold (GMBIT) had a relatively subtle impact on breeding population size, both nationally and regionally (Tables 4, S1). Elasticities were generally somewhat higher in magnitude in East Highlands, Southern Uplands, England and Wales, although the breeding population size in Orkney also showed relatively high response to GMBIT in 2030 (Table S1). As expected, the population breeding on heavily managed grouse moor was negatively influenced by an increase in GMBIT and showed a much larger absolute proportional change than the breeding population nesting in other habitat, where the effect was positive (Table 4). Similarly to most of the parameters investigated, the influence of GMBIT on mean numbers of fledglings was considerably lower than its influence on the national breeding population sizes (Table 4).

**Figure 7:**
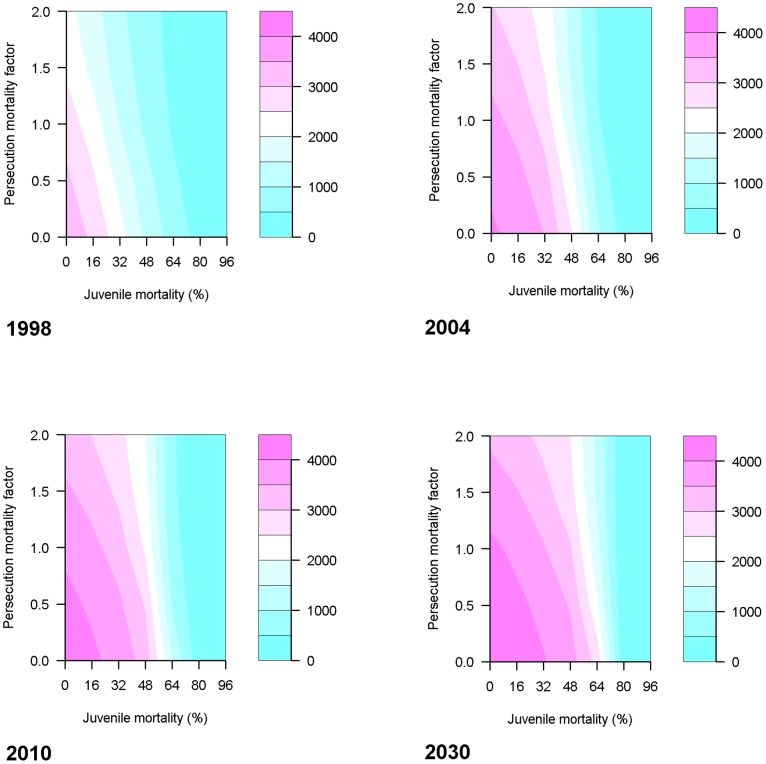
Breeding pair numbers with varying persecution levels and varying juvenile mortality. The average annual national total of breeding pairs for each of the years 1998, 2004, 2010 and 2030, for each combination of the persecution mortality factor (PMF) and juvenile (male and female) mortality (JMmort = JFmort = juvenile mortality). PMF was varied between 0 and 2 inclusive in steps of 0.5. Juvenile mortality was varied between 0 and 96% inclusive in steps of 16%.

PMF and juvenile (male and female) winter mortality showed an interaction, with the national breeding population size decreasing with increases in both mortality parameters (Figure 7). However, at a juvenile mortality of 80% or lower there was little change in population size with either parameter, with population sizes very small and, at 96%, generally going extinct regardless of PMF (Figure 7).

## Discussion

The results presented clearly demonstrate the challenges of accurately replicating trends in real-world populations in the face of large numbers of parameters estimated from published data of varying levels of robustness. At the same time, results such as those regarding the sensitivity of model outputs to juvenile male mortality highlight the importance of accounting for demographic parameters. Such parameters can reverse the entire population trend regardless of the environmental predictors incorporated into the model. Using the baseline scenario with the parameter values based on empirical data we have been able to simulate population dynamics close to empirical observations. Useful insights both into modelling challenges and potential hen harrier dynamics can be gained from analysing the results from the various scenarios presented here.

### 1. Hen harrier population predictions

Like previous modelling attempts [27], [29], the IBM presented here predicted high potential for hen harrier population growth in the north and west of Scotland. The model concurs with predictions for successful breeding conditions in Argyll West and related islands, and for the Outer Hebrides islands ([29] Figures 5–8). The species distribution models (SDMs) of [29] produced presence-absence estimates using predictors based on habitat, climate (which we do not include in our model), topography and potential predators, without incorporating behavioural processes of the hen harrier, and attempting to exclude effects of persecution. The results from the SDMs of [29] were used to predict total potential population size, and although our model showed similar results when run for 150 years (results not shown), long-term predictions were not the main focus of this experiment. This was in part due to the fact that the environmental landscape in the model does not vary over time, while in reality the environment is likely to vary substantially, especially over a period of over 100 years. Hence long-term prediction was not considered appropriate.

Previous modelling attempts have largely focused on mapping potential hen harrier distributions. However, a population may never reach its potential distribution and size – even if this accounts for the presence of environmental and human constraints – if growth is hindered by internal or external factors such as, for example, high natural mortality, high demographic or environmental stochasticity or Allee effects [42], [43]. The results from our model demonstrate the sensitivity of the population growth to mortality, in particular natural juvenile mortality. The regional analyses also showed that while the level of persecution mortality may not be particularly influential at the national scale, it can have a high influence on population growth in specific regions.

The initial sharp declines in numbers of breeding pairs in the East Highlands, Southern Uplands and England simulated by the model in the baseline scenario may relate to the high density of managed grouse moorland in these regions ([27] Figure 2f). This is supported by the high negative elasticities to persecution mortality observed for the numbers of breeding pairs in model results for these regions. For Orkney, the discrepancy between the 1998–2004 recovery seen in census estimates ([32] Table 2) and the continued decline in model results (Figure 5) may be explained by the habitat management that was put in place as a direct result of the decline seen on the islands. This management, which involved agri-environmental methods to increase habitat for hen harriers, was not incorporated into the model and hence the simulated Orkney subpopulation declined to very low numbers (Figure 5).

The continued increase predicted by the model is in contrast to new empirical data, which have shown a national decline between 2004 and 2010 (see [38] and Figure 4). In particular the high population growth predicted for North Highlands is not realised. The 1998–2004 under-prediction for the national total was not fully explained by the sharp decrease in numbers at the beginning of the simulation as percentage increase between 1998 and 2004 continued to be considerably lower in the model than between census estimates. This, together with the inability of the model to capture all intermediate changes in trends, may be due to the static landscape of the model and the static representation of human activity patterns. However, the model over-predicted numbers for 2010, and thus on average might be performing relatively well. Without empirical estimates for future years it is difficult to say how good the model is at short-term predictions.

It is possible that the lower trends might be due to inaccuracies in parameter values. Numbers of breeding pairs corresponded better to earlier census results (1989–2004) when the altitude threshold parameter was not included (results not shown), or when the persecution mortality factor was lowered to between 0 and 0.5 (except for North Highlands). As shown, juvenile male mortality had a high influence on breeding population size. The 64% juvenile male winter mortality used in the baseline scenarios already dramatically reduced the total population size in intermediate and late years.

Unfortunately, robust estimates of juvenile male winter mortality seem to be scarce in the literature. Survival data exist for juvenile females [26], and some population models for harriers only considered female survival [29]. However, these results from our model demonstrate that the male hen harrier population is important to consider when trying to understand hen harrier population dynamics. Results from Orkney have suggested an 86% mortality rate for juvenile males from 0 to 2 years of age [41]. If the survival of yearlings (age 1) to 2 years of age is assumed to be equivalent to the survival of adults (72%; [41]), as it was in our model (78%), the survival rate of juvenile males from 0 to 1 years old would have been 19%, assuming annual survival rates are independent. This is still somewhat lower than the rate we have assumed in the model (36%). With a survival rate of 20% for juveniles, the national population was predicted to decline to less than 200 pairs by 2004 and to ultimately go extinct. Clearly juvenile male winter mortality alone does not explain discrepancies between our model and empirical results. Measurements of mortality on an isolated island of Orkney may also reflect movements of birds to the mainland and hence not be an accurate reflection of mortality across Great Britain. More robust parameter estimates for male survival would help to increase confidence in the model results.

The interaction between persecution mortality and juvenile mortality also indicates that differences in trends may be due to the interplay of several different parameters. The comparison with no non-breeders added at the start of the model run highlights the sensitivity of the model to initial conditions. Another potential factor to explore would be the use of the more detailed regional boundaries (Figure 2B) in the initialisation; this could potentially affect the initial population dynamics of the model. More elaborate elasticity analyses and parameter space explorations can help to extract more information about the interactions between parameters and their significance to the simulated population dynamics [44]–[46]).

A key advantage of the IBM approach is the capacity to include life-history parameters and processes of the study species, to explore which, if any, internal dynamics might be driving population trends. However, this requires robust empirical estimates and an understanding of the uncertainty around these estimates. Even where empirical estimates are somewhat unreliable, IBMs like the hen harrier model we present can provide qualitative results and information regarding what estimates are most crucial for building a realistic representation of the real system. Sensitivity analyses of the model can help to identify the parameters to focus on. Other IBMs of bird species, examining movement and foraging behaviour, have demonstrated the potential of this approach in predicting behaviour in the real world in the presence of relatively basic understanding and empirical data [47], [48]. Such modelling approaches can also allow the confrontation of competing hypotheses about animal behaviour with real-world data [48] and the incorporation of expert knowledge; the latter opening doors to engage stakeholders in conservation conflicts in the modelling and management process which may aid conflict mitigation and resolution [47].

### 2. Model performance

The carrying capacity parameter was introduced to impose restrictions on the numbers of breeding pairs which could share an area and still breed successfully. In reality, factors not explicitly included in the model, such as availability of alternative prey or disease, are likely to limit population growth. Although we introduced no increased mortality as a result of density, the deterrent for breeding in more densely populated areas as a function of a prey availability index was sufficient to reduce breeding population growth rate after year 2031 (results not shown). This is likely to act through a higher proportion of males becoming non-breeders and therefore the proportion of the population which is breeding is reduced, thereby reducing total numbers of offspring relative to population size and hence reducing the population growth rate. The nature of the carrying capacity restriction was based on empirical observations of hen harrier numbers in relation to meadow pipit abundances in the absence of persecution [24]. However, this new regulation was insufficient to prevent the expansion of the hen harrier population well beyond observed numbers.

Because we do not incorporate temporal changes in the environment or in the demographics of hen harriers (such as in individual dispersal or breeding parameters) we cannot expect to replicate temporal trends accurately. The model would be capable of incorporating such data; collecting temporal data on the five environmental variables included here, together with temporal estimates of hen harrier demographic parameters, requires considerable investment of time, resources and personnel, but should be considered for further studies of hen harrier population dynamics.

In addition, we assume that hen harriers are monogamous. However, there is evidence that polygyny is widespread in hen harriers and seems to be unusually prevalent in Orkney [41]. Allowing resident males to acquire more than one female would increase the number of breeding females and might increase population growth, provided that breeding success and average numbers of fledglings per female remain the same. This might not, however, improve the fit of the model predictions to the observed empirical trends.

Despite the above, a process-based model such as the one we present can provide insight into what factors might be the most crucial drivers of population dynamics and where to focus research efforts, rather than just providing potential population sizes. Unlike statistical models based on spatial predictors, individual-based models incorporate demographic parameters whose values can be varied in a systematic, experimental setting to lead to a deeper, mechanistic understanding of system dynamics [49]. It has been argued that understanding, rather than simply predicting, is the most important purpose of modelling [49] and in the light of the model presented here this makes sense; even if short-term trends, such as those between two or three censuses, could be represented accurately, there is no guarantee that long-term predictions would be reliable. However, mechanistic understanding, potentially verified by further empirical observation, can be of more general benefit and can help to focus research efforts where most crucially needed in order to better represent and understand systems.

Methods such as an experimental approach to modelling [49] or the incorporation of modelling as part of an adaptive management framework where empirical data are used to continually refine and update the model to produce better representations of reality [50] can help to reduce model uncertainty. For such an approach, an individual-based model such as the one we have presented is better able to incorporate a range of new information, for example on the behavioural patterns of the species of interest or environmental data, than statistical prediction models or mean-field models lacking spatial predictors.

### 3. Implications

This paper introduces a new hen harrier model which is based on the simulation of behavioural processes as well as statistical predictors of abundance. As such, the model provides a tool incorporating specific behavioural mechanisms used by organisms. This tool can be used in the mapping of spatial and temporal patterns in the abundance of a species, and hence in mapping of conflict areas. Our results illustrate that while historical trends in populations cannot be replicated exactly, a reasonable representation of reality can be achieved when producing a model which is simple enough to allow some inference about the mechanisms of the processes controlling population growth. Further exploration of the model dynamics, perhaps through Bayesian-based sensitivity analyses (see, for example, [46]), can improve understanding of which model parameters and processes are most crucial and hence where data collection efforts should be focused to improve our representation of the hen harrier behaviour and population dynamics, in order to better inform management decisions. Results from our model indicate that juvenile male mortality in particular is a crucial factor in predicting population trends. Persecution mortality estimates are also important. The results also revealed North Highlands as a particular point of disparity between empirical and model results, and further research into the reasons why the apparent potential of this region as hen harrier habitat is not realised may provide important further insight into the threats faced by the hen harrier population in Great Britain.

Representation of human actions and decisions are currently confined to static indices of heather burning as an indication of possible hen harrier persecution. Although requiring more data and assumptions about human behaviour, agent-based models of human stakeholders with explicit decision-making and heterogeneity (see, for example, [51]–[53]) can provide a method for more realistic representations of the conflict between humans and the feedbacks between human attitudes, actions and interactions and the hen harrier population dynamics.

Modelling species such as the hen harrier, which are at the centre of conservation conflicts, presents many challenges. However, within the appropriate framework, models such as the hen harrier model we introduce can provide a useful tool for bringing together information and data on the system and situation, increasing understanding of the system and identifying focal points for future research.

## Supporting Information

Table S1 **Elasticity of the simulated mean number of breeding pairs in selected years to five model parameters, in each region.**
(PDF)

Text S1 **Explanation of the meadow pipit models used to produce the meadow pipit indices.**
(PDF)

## References

[1] Woodroffe R, Thirgood S, Rabinowitz A (2005) People and wildlife: Conflict or coexistence? Cambridge: Cambridge University Press. 497 p.

[2] Baker PJ, Boitani L, Harris S, Saunders G, White PCL (2008) Terrestrial carnivores and human food production: impact and management. Mammal Review 38: 123–166.

[3] Woodroffe R (2000) Predators and people: using human densities to interpret declines of large carnivores. Animal Conservation 3: 165–173.

[4] Lovegrove R (2007) Silent fields: The long decline of a nation’s wildlife. Oxford: Oxford University Press.

[5] Woodroffe R, Thirgood S, Rabinowitz A (2005) The future of coexistence: resolving human-wildlife conflicts in a changing world. In: Woodroffe R, Thirgood S, Rabinowitz A, editors. People and wildlife: Conflict or coexistence? Cambridge: Cambridge University Press. 388–405.

[6] MacDonald D, Service K (2009) Key topics in conservation biology. Wiley-Blackwell.

[7] Sitati NW, Walpole MJ, Smith RJ, Leader-Williams N (2003) Predicting spatial aspects of human-elephant conflict. Journal of Applied Ecology 40: 667–677.

[8] Le Hay G, Clergeau P, Hubert-Moy L (2001) Computerised map of risk to manage wildlife species in urban areas. Environmental Management 27: 451–461. 11148769 10.1007/s002670010161

[9] Tourenq C, Aulagnier S, Durieux L, Lek S, Mesléard F, et al (2001) Identifying rice fields at risk from damage by the greater flamingo. Journal of Applied Ecology 38: 170–179.

[10] Merkle JA, Krausman PR, Decesare N, Jonkel JJ (2011) Predicting spatial distribution of human-black bear interactions in urban areas. The Journal of Wildlife Management 75: 1121–1127.

[11] Treves A, Martin KA, Wydeven AP, Wiedenhoeft JE (2011) Forecasting environmental hazards and the application of risk maps to predator attacks on livestock. BioScience 61: 451–458.

[12] Treves A, Naughton-Treves L, Harper EK, Mladenoff DJ, Rose RA, et al (2004) Predicting human-carnivore conflict: a spatial model derived from 25 years of data on wolf predation on livestock. Conservation Biology 18: 114–125.

[13] Jeltsch F, Müller MS, Grimm V, Wissel C, Brandl R (1997) Pattern formation triggered by rare events: lessons from the spread of rabies. Proceedings of the Royal Society of London Series B 264: 495–503. 9149424 10.1098/rspb.1997.0071 PMC1688393

[14] Bennett VJ, Fernández-Juricic E, Zollner PA, Beard MJ, Westphal L, et al (2011) Modelling the responses of wildlife to human disturbance: an evaluation of alternative management scenarios for black-crowned night-herons. Ecological Modelling 222: 2770–2779.

[15] Grimm V, Berger U, Bastiansen F, Eliassen S, Ginot V, et al (2006) A standard protocol for describing individual-based and agent-based models. Ecological Modelling 198: 115–126.

[16] Jordán F, Scotti M, Priami C (2011) Process algebra-based computational tools in ecological modelling. Ecological Complexity 8: 357–363.

[17] Travis JMJ, Harris CM, Park KJ, Bullock JM (2011) Improving prediction and management of range expansions by combining analytical and individual-based modelling approaches. Methods in Ecology and Evolution 2: 477–488.

[18] De Almeida SJ, Ferreira RPM, Eiras ÁE, Obermayr RP, Geier M (2010) Multi-agent modeling and simulation of an *Aedes aegypti* mosquito population. Environmental Modelling & Software 25: 1490–1507.

[19] Valbuena D, Verburg PH, Bregt AK, Ligtenberg A (2010) An agent-based approach to model land-use change at a regional scale. Landscape Ecology 25: 185–199.

[20] Valbuena D, Bregt AK, McAlpine C, Verburg PH, Seabrook L (2010) An agent-based approach to explore the effect of voluntary mechanisms on land use change: A case in rural Queensland, Australia. Journal of Environmental Management 91: 2615–2625. 20705385 10.1016/j.jenvman.2010.07.041

[21] Bennett VJ, Beard M, Zollner PA, Fernández-Juricic E, Westphal L, et al (2009) Understanding wildlife responses to human disturbance through simulation modelling: a management tool. Ecological Complexity 6: 113–134.

[22] Thirgood S, Redpath S (2008) Hen harriers and red grouse: science, politics and human-wildlife conflict. Journal of Applied Ecology 45: 1550–1554.

[23] Arroyo B, Amar A, Leckie F, Buchanan GM, Wilson JD, et al (2009) Hunting habitat selection by hen harriers on moorland: implications for conservation management. Biological Conservation 142: 586–596.

[24] Redpath SM, Thirgood SJ (1999) Numerical and functional responses in generalist predators: hen harriers and peregrines on Scottish grouse moors. Journal of Animal Ecology 68(5): 879–892.

[25] Thirgood SJ, Redpath SM, Haydon DT, Rothery P, Newton I, et al (2000) Habitat loss and raptor predation: disentangling long- and short-term causes of red grouse declines. Proceedings of the Royal Society of London Series B 267 (1444): 651–656. 10.1098/rspb.2000.1051 PMC1690583 10821608

[26] Etheridge B, Summers RW, Green RE (1997) The effects of illegal killing and destruction of nests by humans on the population dynamics of the hen harrier *Circus cyaneus* in Scotland. Journal of Applied Ecology 34: 1081–1105.

[27] Anderson BJ, Arroyo BE, Collingham YC, Etheridge B, Fernandez-De-Simon J, et al (2009) Using distribution models to test alternative hypotheses about a species’ environmental limits and recovery prospects. Biological Conservation 142: 488–499.

[28] Potts GR (1998) Global dispersion of nesting hen harriers *Circus cyaneus*; implications for grouse moors in the U.K. Ibis. 140: 76–88.

[29] Fielding A, Haworth P, Whitfield P, McLeod D, Riley H (2011) A Conservation Framework for Hen Harriers in the United Kingdom. JNCC Report 441. Joint Nature Conservation Committee, Peterborough.

[30] New LF, Buckland ST, Redpath S, Matthiopoulos J (2011) Hen harrier management: insights from demographic models fitted to population data. Journal of Applied Ecology 48: 1187–1194.

[31] Grimm V, Berger U, DeAngelis DL, Polhill JG, Giske J, et al (2010) The ODD protocol: a review and first update. Ecological Modelling 221: 2760–2768.

[32] Sim IMW, Dillon IA, Eaton MA, Etheridge B, Lindley P, et al (2007) Status of the Hen Harrier *Circus cyaneus* in the UK and Isle of Man in 2004, and a comparison with the 1988/89 and 1998 surveys. Bird Study 54: 256–267.

[33] Risely K, Noble DG, Baillie SR (2008) The breeding bird survey 2007. BTO Research Report 508. Thetford: British Trust for Ornithology.

[34] Risely K, Noble DG, Baillie SR (2009) The breeding bird survey 2008. BTO Research Report 537. Thetford: British Trust for Ornithology.

[35] White RM, Fischer A, Marshall K, Travis JMJ, Webb TJ, et al (2009) Developing an integrated conceptual framework to understand biodiversity conflicts. Land Use Policy 26: 242–253.

[36] Countryside Information System. Available: http://www.ceh.ac.uk/products/software/cehsoftware-cis.htm. Accessed 2014 Sep 1.

[37] Arroyo B, Leckie F, Amar A, McCluskie A, Redpath S (2014) Ranging behaviour of hen harriers breeding in Special Protection Areas in Scotland. Bird Study 61(1): 48–55.

[38] Hayhow DB, Eaton MA, Bladwell S, Etheridge B, Ewing SR, et al (2013) The status of the hen harrier, *Circus cyaneus*, in the UK and Isle of Man in 2010. Bird Study 60(4): 446–458.

[39] Sharrock JTR (1976) The atlas of breeding birds of Britain and Ireland. Calton: Poyser. 479 p.

[40] Redpath S, Madders M, Donnelly E, Anderson B, Thirgood S, et al (1998) Nest site selection by hen harriers in Scotland. Bird Study 45(1): 51–61.

[41] Picozzi N (1984) Sex ratio, survival and territorial behaviour of polygynous Hen harriers *Circus c. cyaneus* in Orkney. Ibis 126: 356–365.

[42] Stephens PA, Sutherland WJ (1999) Consequences of the Allee effect for behaviour, ecology and conservation. Trends in Ecology and Evolution 14(10): 401–405. 10481204 10.1016/s0169-5347(99)01684-5

[43] McCaffery RM, Eby LA, Maxell BA, Corn PS (2014) Breeding site heterogeneity reduces variability in frog recruitment and population dynamics. Biological Conservation 170: 169–176.

[44] Cariboni J, Gatelli D, Liska R, Saltelli A (2007) The role of sensitivity analysis in ecological modelling. Ecological Modelling 203: 167–182.

[45] Saltelli A, Annoni P (2010) How to avoid a perfunctory sensitivity analysis. Environmental Modelling & Software 25: 1508–1517.

[46] Parry HR, Topping CJ, Kennedy MC, Boatman ND, Murray AWA (2013) A Bayesian sensitivity analysis applied to an Agent-based model of bird population response to landscape change. Environmental Modelling & Software 45: 104–115.

[47] Aben J, Strubbe D, Adriaensen F, Palmer SCF, Travis JMJ, et al (2014) Simple individual-based models effectively represent Afrotropical forest bird movement in complex landscapes. Journal of Applied Ecology 51(3): 693–702.

[48] Cortés-Avizanda A, Jovani R, Donázar JA, Grimm V (2014) Bird sky networks: how do avian scavengers use social information to find carrion? Ecology 95(7): 1799–1808. 25163114 10.1890/13-0574.1

[49] Grimm V (1999) Ten years of individual-based modelling in ecology: what have we learned and what could we learn in the future? Ecological Modelling 115: 129–148.

[50] Bunnefeld N, Hoshino E, Milner-Gulland EJ (2011) Management strategy evaluation: a powerful tool for conservation? Trends in Ecology and Evolution 26(9): 441–447. 21680051 10.1016/j.tree.2011.05.003

[51] An L (2012) Modelling human decisions in coupled human and natural systems: Review of agent-based models. Ecological Modelling 229: 25–36.

[52] Bosquet F, Le Page C (2004) Multi-agent simulations and ecosystem management: a review. Ecological Modelling 176: 313–332.

[53] Filatova T, Verburg PH, Parker DC, Stannard CA (2013) Spatial agent-based models for socio-ecological systems: Challenges and prospects. Environmental Modelling & Software 45: 1–7.

