# Supplementary material for: Modelling Hen Harrier Dynamics to Inform Human-Wildlife Conflict Resolution: A Spatially-Realistic, Individual-Based Approach
Source: PLoS One. 2014 Nov 18;9(11):e112492. doi: 10.1371/journal.pone.0112492 (PMC4236102; doi:10.1371/journal.pone.0112492)
Supplement: Table S1 — Elasticity of the simulated mean number of breeding pairs in selected years to five model parameters, in each region. (PDF) [file pone.0112492.s001.pdf]

Table S1. Elasticity of the simulated mean number of breeding pairs in selected years to five model parameters, in each region.

Region	Year	Parameter				
		Altitude threshold MAXALT (m)	Heath threshold MINHEATH (ha)	Persecution mortality factor PMF	Juvenile male mortality JMmort (%)	Grouse moor burning index threshold GMBIT
Orkney	1998	-0.265	0.051	0.000	0.028	0.020
	2004	0.194	-0.607	-0.239	-0.941	-0.023
	2010	1.075	-0.208	-0.422	-1.969	-0.047
	2030	0.000	-0.519	-0.833	-4.183	0.279
Hebrides	1998	-0.308	0.415	-0.407	-2.181	-0.054
	2004	0.057	0.876	-0.794	-2.999	-0.096
	2010	0.239	0.776	-1.101	-3.599	-0.049
	2030	0.280	0.184	-0.172	-4.231	-0.007
NH	1998	0.039	-0.128	0.086	-1.807	0.026
	2004	0.228	-0.049	-0.252	-2.977	0.036
	2010	0.390	-0.076	-0.209	-3.507	0.044
	2030	0.524	-0.159	-0.122	-3.482	0.026
WH	1998	-0.121	-0.629	-0.302	-1.916	0.056
	2004	-0.244	-0.662	-0.324	-2.669	0.049
	2010	0.036	-0.601	-0.386	-3.224	0.065
	2030	0.260	-0.542	-0.213	-3.427	0.023
EH	1998	0.103	-0.188	-1.340	-0.517	0.140
	2004	0.167	-0.390	-0.676	-2.360	0.192
	2010	0.249	-0.600	-0.848	-3.130	0.219
	2030	0.818	-0.459	-0.750	-3.923	0.120
SU	1998	-0.169	-0.167	-1.413	-0.536	0.101
	2004	-0.608	-0.370	-1.988	-1.339	0.110
	2010	-0.667	-0.920	-1.741	-2.320	0.125
	2030	-0.070	-0.473	-1.255	-3.670	0.143
England	1998	-0.523	-0.511	-3.121	4.090	-0.093
	2004	-2.550	0.900	-2.455	1.450	0.136
	2010	-1.731	-1.324	-3.656	-3.076	-0.063
	2030	-1.929	-0.643	-3.857	-4.028	1.500
Wales	1998	0.126	-0.269	-1.117	-0.525	-0.001
	2004	-0.258	0.614	-1.006	-1.358	0.125
	2010	-0.561	-0.088	-1.502	-2.446	0.235
	2030	-0.476	-0.972	-1.632	-3.659	0.275
ET	1998	-0.250	0.623	-2.747	3.093	-0.119
	2004	-0.900	0.686	-1.089	0.107	0.067
	2010	-0.633	0.000	-1.023	-2.990	0.088
	2030	0.635	0.191	-0.832	-3.724	0.218
WT	1998	-0.121	-0.128	-0.938	-0.476	-0.031
	2004	-0.142	0.604	-0.808	-1.663	0.066
	2010	-0.326	0.307	-0.814	-2.660	0.152
	2030	-0.213	-0.630	-0.940	-3.319	0.210

Region codes are as follows: NH – North Highlands; WH – West Highlands; EH – East Highlands; SU – Southern Uplands; England – England (census breeding pair counts only); Wales – Wales (census breeding pair counts only); ET – All of England; WT – All of Wales.
