# Supplementary material for: Modelling Hen Harrier Dynamics to Inform Human-Wildlife Conflict Resolution: A Spatially-Realistic, Individual-Based Approach
Source: PLoS One. 2014 Nov 18;9(11):e112492. doi: 10.1371/journal.pone.0112492 (PMC4236102; doi:10.1371/journal.pone.0112492)
Supplement: Text S1 — Explanation of the meadow pipit models used to produce the meadow pipit indices. (PDF) [file pone.0112492.s002.pdf]

Text S1. Explanation of the meadow pipit models used to produce the meadow pipit indices.

Data sources and preparation

MP abundance data

Breeding Bird Survey (BBS) data on MP abundance were obtained for the period 1994-2007. These comprised the total number recorded on each square at the early (April-May) and late (June-July) visits. As zero counts are not entered by volunteer BBS recorders, a complete set of square-year-visit records was also provided, from which zero counts could be deduced. The number of years recorded varied greatly between squares, and in some cases, there were gaps in the square's record. As Redpath and Thirgood [1] estimated MP abundance during June, BBS data from the late visit only were used. A count was rejected if there was a strong possibility that the late visit was actually missing (i.e. high early visit count and zero late visit count in the same year); this could arise if the observer omitted to enter MP data for the late visit, or genuinely if the square held pipits on migration during spring, but not as a breeding species.

Land cover data

Summary data from Land Cover Map 2000 (LCM) at the scale of 1 km^2 squares were obtained under licence from the Centre for Ecology and Hydrology. These data were at the Broad Habitat level, and the total area (ha) of each of the following classes was extracted: broadleaved woodland, conifer woodland, arable, improved grassland, acid grassland, neutral grassland, calcareous grassland, bracken, dense dwarf shrub heath, open dwarf shrub heath, bog, montane and inland water. The mean altitude of each square was extracted from the Countryside Information System (CIS).

Data preparation

The areas of broadleaf and conifer woodland were combined into a single woodland class, as were the heath classes, and the areas of acid, neutral and calcareous grassland into a single rough grassland class. To allow for possible curvilinear relationships, quadratic terms were derived for easting, northing, altitude, rough grassland, heath and bog[1].

The mean MP count over all years was calculated for each square in Great Britain (GB). Squares having fewer than 3 years' count data were discarded (869 out of 4572 squares, leaving a total of 3703 squares). The data were then split randomly into a parameter-estimation set (comprising approximately 2/3 of the squares; n = 2493 squares) and a validation set (approximately 1/3; n = 1210 squares). The mean counts were heavily overdispersed, as almost 70% of English squares (3212 squares) never recorded a MP. Thus there could be merit in adopting a two-stage model structure: (i) a model to predict MP presence and (ii) a model to predict abundance if present.

[1] These preliminary analyses were actually conducted on habitat data extracted from CIS, which differed in some squares from the CEH summary data obtained for the main analyses.

Correcting for biased under-prediction of abundance

Predicting abundance by back-transformation from the predictions of a model in which the response variable is log-transformed will always lead to an under-estimate of the true value. Beauchamp and Olson [2] have shown that adding half the root mean square error of the regression model to a predicted value prior to back-transforming (i.e. $y = exp[\mu + \sigma^2/2]$ rather than $y = exp[\mu]$), whilst not unbiased, is a close approximation to the unbiased estimate. However, adapting this method to a generalised linear model (GLM) assuming Poisson-distributed residuals for a one-stage model can led to a high degree of over-estimation. This is presumably because the mean square error in the Poisson model is proportional to the mean, rather than independent of it as in a normal-error model (in fact, it could be being made far worse by over-dispersion).

Fitted models for Great Britain

The models

Stage 1: The presence of MP was fitted to a logistic model using a logit link function and binomial error term (SAS procedure GENMOD).

Stage 2:

A) The mean MP abundance in squares where present was fitted to a GLM using a log link function and Poisson error term (SAS procedure GENMOD; the scale parameter was estimated by the square root of the deviance/d.f. (degrees of freedom), as there remained some overdispersion in the data).

B) As (A) but the predicted abundances where present were corrected for under-prediction.

C) The mean MP abundance in squares where present was fitted to a generalised additive model (GAM) assuming a Normal distribution and identity link function (the GAM procedure did not allow a log link function as the dependent variable was not in integers).

Explanatory variables

The land cover categories and quadratic terms as described above in “Land cover data” and “Data preparation” were used as explanatory variables, together with the easting, northing and mean altitude (see “Data preparation”). For the two models other than the generalised additive model (GAM), all possible explanatory variables were initially included in the model and the non-significant terms dropped sequentially on the basis of their Wald type 3 X^2 values until the best-fitting model was reached on the basis of the AIC value. For the GAM, possible explanatory variables were added sequentially to the model, initially including a cubic spline for the variable. Spline terms were omitted if there was no justification for them; in this case only the linear term for the variable would be included, and this also was rejected if it was clearly not a significant predictor of MP abundance. Quadratic terms were not included as the spline should allow for non-linear relationships.

Summary of results

Model	Estimation set Correlation[a]	Validation Set Correlation[b]	Highest Predicted abundance
2 stage: presence, abundance where present (A) (GENMOD)	0.71 (2nd stage only)	0.78[c]	59
2 stage: presence, abundance where present, adjusted (B) (GENMOD)	0.71 (2nd stage only)	0.78[c]	119
2 stage: presence, abundance where present (C) (GAM)	0.78 (2nd stage only)	0.76	43.5

[a]The Pearson correlation between predicted and actual abundance on the raw data scale for the parameter-estimation dataset

[b]The Pearson correlation between predicted and actual abundance on the raw data scale for the validation dataset

[c]The correlations are the same because the same model was used, with (B) then corrected for under-prediction through log-transformation

Discussion

All models had a tendency to under-predict high mean counts as a result of the link function used. The results from the GLM are known to be biased without correction for under-prediction. However, the adjustment made in the case of the GLM resulted in unrealistically high maximum MP abundances. The GAM was considered the overall best fit due to its higher correlation with the estimation abundances and relatively high correlation with the estimation data set.

References

1. Redpath SM, Thirgood SJ (1999) Numerical and functional responses in generalist predators: hen harriers and peregrines on Scottish grouse moors. Journal of Animal Ecology 68: 879-892.
2. Beauchamp JJ, Olson JS (1973) Corrections for bias in regression estimates after logarithmic transformation. Ecology 54: 1403-1407.
